# Business Process Model and Notation and openEHR Task Planning for Clinical Pathway Standards in Infections: Critical Analysis

**DOI:** 10.2196/29927

**Published:** 2022-09-15

**Authors:** Natalia Iglesias, Jose M Juarez, Manuel Campos

**Affiliations:** 1 Instituto de Investigación de Tecnologías de la Información y las Comunicaciones Orientadas University of Murcia Murcia Spain; 2 Instituto Murciano de Investigación Biosanitaria - Arrixaca Murcia Spain

**Keywords:** openEHR task planning, business process model and notation, BPMN, clinical pathways, catheter-related bloodstream infection, CR-BSI, clinical guidelines

## Abstract

**Background:**

Clinical pathways (CPs) are usually expressed by means of workflow formalisms, providing health care personnel with an easy-to-understand, high-level conceptual model of medical steps in specific patient conditions, thereby improving overall health care process quality in clinical practice. From a standardized perspective, the *business process model and notation* (BPMN), a widely spread general-purpose process formalism, has been used for conceptual modeling in clinical domains, mainly because of its easy-to-use graphical notation, facilitating the common understanding and communication of the parties involved in health care. However, BPMN is not particularly oriented toward the peculiarities of complex clinical processes such as infection diagnosis and treatment, in which time plays a critical role, which is why much of the BPMN clinical-oriented research has revolved around how to extend the standard to address these special needs. The shift from an agnostic, general-purpose BPMN notation to a natively clinical-oriented notation such as *openEHR Task Planning* (TP) could constitute a major step toward clinical process improvement, enhancing the representation of CPs for infection treatment and other complex scenarios.

**Objective:**

Our work aimed to analyze the suitability of a clinical-oriented formalism (TP) to successfully represent typical process patterns in infection treatment, identifying domain-specific improvements to the standard that could help enhance its modeling capabilities, thereby promoting the widespread adoption of CPs to improve medical practice and overall health care quality.

**Methods:**

Our methodology consisted of 4 major steps: identification of *key features* of infection CPs through literature review, clinical guideline analysis, and BPMN extensions; analysis of the presence of *key features* in TP; modeling of relevant process patterns of catheter-related bloodstream infection as a case study; and analysis and proposal of extensions in view of the results.

**Results:**

We were able to easily represent the same logic applied in the extended BPMN-based process models in our case study using *out-of-the-box* standard TP primitives. However, we identified possible improvements to the current version of TP to allow for simpler conceptual models of infection CPs and possibly of other complex clinical scenarios.

**Conclusions:**

Our study showed that the clinical-oriented TP specification is able to successfully represent the most complex catheter-related bloodstream infection process patterns depicted in our case study and identified possible extensions that can help increase its adequacy for modeling infection CPs and possibly other complex clinical conditions.

## Introduction

### Background

The interoperability of clinical information is a major issue that hinders data exchange and knowledge reuse between clinical institutions [1]. An important type of clinical information is the representation of institution-specific care protocols, known as clinical pathways (CPs), defined as “task orientated care plans which detail essential steps in the care of patients with a specific clinical problem and describe the patient’s expected clinical course” [2]. This definition has been reviewed in the studies by De Bleser et al [3] and Vanhaecht et al [4] and further refined in the study by Kinsman et al [5] through five criteria: “(1) a structured multidisciplinary plan of care; (2) used to channel the translation of guidelines or evidence into local structures; (3) detailed the steps in a course of treatment or care in a plan, pathway, algorithm, guideline, protocol or other inventory of actions; (4) had timeframes or criteria-based progression (that is, steps were taken if designated criteria were met); and (5) aimed to standardise care for a specific clinical problem, procedure or episode of healthcare in a specific population.” CPs often rely on medical evidence expressed in clinical guidelines (CGs), which standardize abstract best practices for the diagnosis and treatment of specific medical conditions based on both medical evidence and expert consensus to ultimately improve the quality and uniformity of care, describing the strict temporal order in which clinical work needs to be carried out. For this purpose, many clinical task-oriented tools have been used over the years, such as Asbru, GLIF, GLARE, PROforma, EON, or GUIDE [6], but none of them seem to have reached the popularity of general-purpose process management tools such as the *business process model and notation* (BPMN) from the Object Management Group (OMG) [7], probably because of their complexity and narrow use limited to high-technology institutions with enough financial and technical means. In the last years, BPMN, which is widely used in other industry domains, has also emerged in clinical domains as a process management standard. Many studies describe the use of BPMN to represent clinical processes to improve efficiency or serve as a basis for the development of clinical decision support systems, which require seamless integration between electronic health record (EHR) data, decisions, and the specific workflow of a medical institution [8-12]. Although BPMN has proven to be effective in representing clinical processes, the transition to process execution in real clinical institutions is still scarce [13]. Indeed, although BPMN can effectively help clinicians visually understand clinical processes and detect possible inefficiencies, the implementation of otherwise complex, stepwise clinical workflows in an ordered manner is not easy to accomplish as temporal interrelations between tasks are crucial. In the last years, new clinical-oriented process representation standards have emerged that address the specific needs of clinical workflows, such as openEHR Task Planning (TP) [14]. Our work is based on the hypothesis that the clinical-oriented business process management (BPM) tool TP provides a more adequate way to represent CPs, bridging the gap between abstract CG logic and clinical workflow and offering essential tools for the representation of complex health care processes such as infection treatment. In our research, we analyze how TP can represent complex infection CPs taking as a case study the catheter-related bloodstream infection (CR-BSI) CGs developed by the Johns Hopkins Hospital (JHH) Antimicrobial Stewardship Program, an international *gold standard* that includes recommendations aiming to standardize clinical practice around antibiotic prescription, thereby helping minimize antibiotic resistance [15].

The structure of this paper is as follows: in the *Introduction* section, we explain the background; in the *Methods* section, we illustrate the methodology used; in the *Results* section, we describe our experiments; and, in the *Discussion* section, we discuss the relevance and limitations of the results.

### Related Work

The best up-to-date evidence-based clinical knowledge is usually expressed in CGs, defined as “systematically developed statements to assist practitioner and patient decisions about appropriate healthcare for specific clinical circumstances” [16], often used in a paper-based mode. Many studies have successfully represented CG knowledge using different notations, such as the procedural medical-oriented *Arden Syntax*, rule-based systems such as *Drools*, guideline definition languages (*Graphic Language for Interactive Design [GLIDE]* and the *openEHR Guideline Definition Language*), or *Semantic Web* rule systems such as *SPARQL Inferencing Notation* (SPIN) or *Shapes Constraint Language* (SHACL) [17]. However, knowledge representation technologies do not suffice to represent a specific patient evolution over time, which has led to the development of medical-oriented task-based systems such as *PROforma* [18], *Asbru* [19], or *Prodigy* [20], which have been successfully used in many clinical scenarios but are effectively limited to a few medical institutions owing to the high costs and efforts associated with their implementation. This has favored the introduction in the last years of popular, easy-to-use, general-purpose BPM standards in the health care landscape as a working alternative to complex, medical task-oriented knowledge systems. However, modeling specific constraints in clinical processes remains a challenge because of the intrinsic domain complexity. Much research has been conducted over the years to eliminate or compensate for the temporal shortcomings of generic BPM notations, such as with *Petri nets* (a graphical workflow formalism for conceptual modeling of distributed systems [21,22]), *Process Mining for Healthcare* [23], or improved integration with data or decision support [9,10,24].

### Clinical Context

CR-BSI is a highly recurring infection and a major cause of morbidity and costs in hospitals. The leading cause of CR-BSI is gram-positive bacteria present in intravascular catheters, especially the coagulase-negative *Staphylococcus* species, which must be treated with a multidisciplinary approach based on catheter removal or catheter salvage combined with an antimicrobial *lock therapy*. Only in the United States, >150 million intravascular catheters are purchased by hospitals each year [25,26]. In addition, >250,000 intravascular CR-BSI cases happen each year, with an attributed mortality rate of 12% to 25% [27]. Of these, approximately 80,000 CR-BSI cases take place in intensive care units, where “more than 15 million central vascular catheter (CVC) days occur each year” [28]. This happens in a context of insufficient research on new antibiotics and limited supply of the existing ones, which narrows down the possible therapies for highly recurring bacterial infections, many of which are still susceptible to generic antibiotics with lower toxicity levels and lower risk of resistance development [29]. The treatment options for CR-BSI depend on the microorganism causing the infection, which determines the type of antibiotic and the time, frequency, and dosage of its intake. Furthermore, the type of catheter, the way it is handled, the duration of its placement, and the patient conditions codetermine the risk of a hospital-acquired CR-BSI, increasing the length of hospital stay and mortality figures especially in patients who are critically ill and whose catheter is not removed [30]. Therefore, specific CGs have been developed for health care staff to prevent the development of CR-BSI and issue recommendations regarding the most suitable course of treatment. The JHH CGs, based on current literature, Infectious Diseases Society of America national guidelines [31], and JHH medical evidence, cover the entire infection course from diagnosis through antibiotic treatment and assist clinicians in the selection of the optimal antibiotic therapy in an attempt to fight antibiotic resistance and still remain effective [15].

### BPMN Process Formalism

BPMN is a mature, general-purpose BPM graphical representation and ISO standard developed by OMG based on an unstructured graph-oriented language combined with features from other workflow languages that can be represented using *Petri nets* [32]. In BPMN, a process is a free sequence of activities or events ordered in a sequence flow and connected through split or merge gateways that redirect the flow into one or multiple paths. This standard has been widely used by business process managers in many different application domains owing to its simplicity. Despite not being specifically designed for clinical processes, BPMN has proven its value in the health care domain to represent CPs, allowing for an easy-to-understand representation of CP recommendations [12,13,33,34]. However, its use in clinical domains is still scarce owing to the complexity of clinical processes and organizations, the critical relevance of care protocols, the difficulties in handling temporal constraints, and the uncertainty surrounding patient evolution and treatment effectiveness [13]. Furthermore, the integration of BPMN process models with EHR data is usually modeled using Unified Modeling Language [35] to address the BPMN shortcomings in that regard [24]. In addition, BPMN provides a limited set of capabilities for modeling temporal constraints [34,36] as the standard lacks out-of-the-box temporal semantics. Therefore, many extensions of the BPMN metadata model have been proposed over the years to address the specific demands of clinical processes [11,34,36-44].

### openEHR TP

openEHR is an initiative of the openEHR Foundation working closely with the European Committee for Standardization (CEN), the International Organization for Standardization (ISO), Health Level 7 (HL7), OMG, and other organizations on EHR and clinical modeling standards that provides an archetype-based standard data model for EHR systems designed with interoperability in mind [45]. In 2016, the openEHR Foundation released a new clinical-oriented workflow management standard, the TP specification, that includes a Visual Modeling Language (TP-Visual Modeling Language [TP-VML]) backed up by formal semantics, which ultimately would allow for the automatic translation of graphical workflow models into executable models [14,46]. TP complements openEHR by allowing modeling orders and actions in the future, structured as work or task plans, as well as their eventual execution in distributed environments, improving features partially present in other workflow languages (BPMN, Yet Another Workflow Language [YAWL] [47], Case Management Model and Notation [CMMN] [48], or Decision Model and Notation [DMN] [49]). The TP engine executes both TP work plans and openEHR Decision Language (DL) rules [50]. Moreover, TP allows for the specification of complex times in medication orders (eg, “3 times a day before meals” [51]), and it is conceived as a clinical process navigator, empowering users to perform ad hoc modifications at the time of execution to reflect real-time changes and supporting auditing, reporting, and billing to analyze task performance, execution, and eventual deviations from the original plan. Although openEHR has a wide coverage as a research area [52-54], the TP specification has been used in fewer studies [55-57] and is still under development. The contributions of our work to support our hypothesis of the native suitability of clinical-oriented BPM formalisms for infection CPs are as follows: (1) identification of key features required for modeling infection CPs, as laid out in the literature, the JHH CGs, and the successive BPMN extensions; (2) theoretical analysis of how TP addresses the identified key features; (3) a case study to empirically show the native TP adequacy by modeling typical CR-BSI infection patterns using TP; and (4) analysis and proposal of potential extensions of the current version of TP for improved modeling of infection CPs and possibly of other complex CPs.

## Methods

### Overview

**Figure 1:**
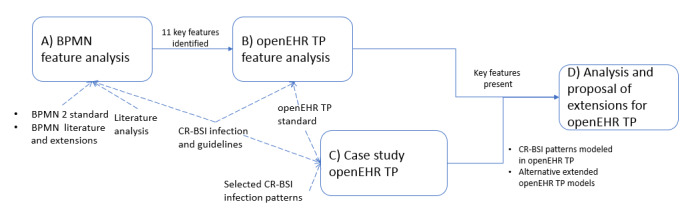
Methodology followed. BPMN: business process model and notation; CR-BSI: catheter-related bloodstream infection; TP: Task Planning; TP-VML: Task Planning Visual Modeling Language.

Our research aimed to explore and identify *key features* to represent infection CPs to then analyze how they are present in the TP standard. The analysis can lead to a potential list of benefits and possible improvements for the representation of infection CPs using TP. For this purpose, in this study we adopt the methodology illustrated in Figure 1.

### Step A

We identified *key features* required for modeling infection CPs by reviewing scientific literature, BPMN extensions in clinical settings, JHH CGs, and an *extended BPMN*-based model of JHH CR-BSI. The identified *key features* formed a basic set of requirements that served as a starting point to analyze the theoretical adequacy of TP for the representation of infection CPs.

### Step B

We conducted a theoretical analysis of the presence of the previously identified *key features* in the TP specification by performing an in-depth evaluation of the current version of TP to understand how these capabilities were addressed by the standard. When possible, a one-to-one comparison of both standards’ capabilities was performed, which could help identify possible improvements. In addition, when new, potentially interesting TP capabilities were detected, we discussed their relevance.

### Step C

We conducted an empirical test of the suitability of the current version of TP for conceptual modeling of infection CPs. To that end, we modeled typical infection treatment patterns from the JHH CR-BSI CGs using the same logic used in the extended BPMN version [44]. When opportunities for improvements were detected, we proposed alternative TP models.

### Step D

Taking the output of steps B and C, we proceeded with the analysis of benefits and potential enhancements to the current version of the TP standard and made a proposal of possible extensions to improve the representation of infection CPs. For this purpose, we followed the steps of the methodology for BPMN extensions laid out in the study by Braun and Schlieter [58], which we consider adequate for TP.

## Results

### Step A: Identification of Key Features for Infection CPs

BPMN has been extended over the years with domain-specific concepts to meet the main requirements of process representations in clinical domains. Therefore, an analysis of the BPMN missing capabilities through its extensions in clinical settings is essential to identify *key features* that any CP representation should fulfill. Furthermore, any language for modeling CPs should contain (1) concepts for medical business process modeling (*patient state*, *treatment step*, *decision,* or *process flow*) plus the ability to integrate information objects and responsibilities, (2) indefinite order relations as well as compulsory parallel relations between treatment steps and iterating treatment steps, (3) the evidence class of any recommendation and decision and a link to the source of the evidence, and (4) temporal dependencies and explicit time events [58].

The *key features* proposed in this study were derived from an analysis of existing literature selected based on quality and content regarding (1) BPM extensions in clinical domains affecting the representation of, among others, time, resources, and coordination of CPs by multidisciplinary teams working together in a care process [8,36-39,41,58-63]; (2) review studies of BPMN extensions both in clinical and nonclinical domains [13]; and (3) analysis of JHH CG features for infection management [15,43,44], such as parallel work, synchronization with EHR data, or uncertainty in the course of an infection treatment. Although broadly inclusive, our criteria were proposed as a set of minimum requirements to represent infection CPs. First, the *well-structuredness* of a process conceptual model helps increase its legibility and understanding by clinical staff, thereby enabling the detection and analysis of possible process improvements. In addition, it is a requirement to avoid deadlocks and inconsistencies in and between processes, thereby facilitating the future maintenance and evolution of the process representation. *Process modularity* allows for the decomposition of large, complex clinical processes with many involved parties in their constituent subprocesses, thereby allowing for increased readability, process reuse, and faster model building and verification of soundness across the organization. *Events* allow for conceptual modeling of facts that take place at the time of execution, the occurrence of which is possibly unknown at the time of design. Events deal with uncertainty and are always present in the treatment of infections, whose course depends on factors unknown at the time of design, such as the patients’ eventual response to medication. *Parallel execution of tasks* optimizes execution time to promptly react to clinical happenings as clinical institutions consist of complex multilayered organizational units, possibly externalized and interacting with each other. *Task duration* allows for the expression of a constraint, a deadline, or simply information to all process actors. For example, a blood test can take a minimum of 1 day and a maximum of 2 days to complete. If purely informational, workflow execution should not wait for this task to complete but, if meant as a constraint, the model should include semantics to hold the execution of subsequent tasks. The time distance between nonconsecutive tasks can be specified using *relative time constraints* such as edge duration between the starting or ending instant of a predecessor task A and the starting or ending instant of a successor task B. These constraints are determined by best practices or the institution’s average figures to define a minimum or maximum waiting time when dispatching work to other organizational units. Synchronization between subprocesses is ultimately reduced to the synchronization between their inner tasks. *Use of resources* is relevant in infection CPs as catheters are a main cause of infection in hospitalized patients, requiring both *catheter lock therapy (CLT)* and *systemic therapy (ST)* applied repeatedly and alternatively but not simultaneously through the existing catheter, which must therefore be used in exclusive mode by the executing task. *Multiple instances of a task* might be required to unfold repeatable tasks such as therapy or follow-up tasks, although, in many cases, their multiplicity level is unknown at the time of design and can only be determined at the time of execution (either by the user or by the process logic) as it depends on the clinical evolution of a patient or the adequacy of the prescribed treatment. *Delays between iterations of looping activities*, executed as long as a loop condition evaluates to *true*, allow for the expression of things such as “Vancomycin every 8 hours during 7 days” [7]. Furthermore, the *integration of clinical* data is useful for reviewing the availability of process-required data, their location, or the detection of inconsistencies or potential improvements. *Data* are used for input or output to make decisions, initiate a process, coordinate tasks, or inform process actors, whereas *data stores* allow for data persistence beyond the execution of a process model. In addition, in infection CPs, a close relationship between EHR information and model metadata is required to be able to dynamically adapt to clinical happenings. Finally, *overrides at execution time*, either by the user or the system’s logic, are required to deal with unexpected happenings during the course of an infection, which can unleash relevant changes in the care process.

### Step B: Identification of Corresponding TP Features for Infection CPs

The TP specification is natively oriented toward the conceptual modeling of clinical processes, thus theoretically providing out-of-the-box capabilities that are present in BPMN only after successive extensions. In this section, we analyze how TP supports the *key features* identified in the previous section. First, *well-structuredness* is a *built-in* feature in the TP standard semantics, which impose implicit restrictions on a process model. *Process modularity* is achieved in TP by the intrinsic grouping of reusable pieces of work in work plans, task plans, and, optionally, subplans for fine-grained work details. TP *events* regulate the temporal behavior of TP tasks or task groups by means of *task-waits* that prevent task execution until facts occur that satisfy a wait condition, such as the occurrence of a task transition or changes in global tracked variables. *Parallel execution of tasks* can be achieved in TP using the *execution type* attribute of the *plan item* top-level class. In addition, *conditional decision* structures exist to represent *if-elseif-else*, *case*, or *event*-*based* conditions, and the optional *concurrency-mode* attribute defines 4 possible states of a task group during and after the execution of its conditional paths. However, *task duration,* either as constraint or merely informative, does not exist in the current version of TP. When duration is meant as a constraint, TP implements this behavior through *task-waits* associated with events instead. *Relative time constraints between tasks* are managed in TP through *task-waits* for both deterministic and nondeterministic events combined with the use of *dispatchable* tasks. *Resources* are minimally represented in TP through *Resource_Participation* objects required for task execution. The allocation and tracking of workers to a task or task group is done at the time of execution as part of the *materialized* model (ie, the M* classes, which are only minimally specified in the current TP version). *Multiple instances of a TP task* can be dynamically generated at the time of execution if they are marked as *repeatable* in the process model by the optional *repeat-spec* attribute, which specifies the minimum and maximum number of iterations, and an optional *terminate condition* to exit the *repeat* loop. Each iteration is unrolled into literal sequential copies in the *materialized* image of the work plan. *Delays between iterations of looping tasks* or *task groups* are possible with the optional *period* attribute of a *repeat-spec* associated with a repeatable task or task group to be able to express things such as *daily every 8 hours,* acting as a spacer between the execution of the literal copies of each iteration. Furthermore, *clinical data integration* is indirectly achieved through the *capture data set* attribute, a data set template or form via which data can be introduced during task execution. As TP is part of the openEHR standard, data references are encapsulated using *archetypes*, so the *template-id* attribute specifies the *archetype human-readable identifier* (HRID) to be used, triggering the population of a data set [64]. Finally, *overrides at execution time* are *built-in* in TP, which by default assumes that, at the time of execution, users can have information that is unknown to the system for any number of reasons. The designed process model is performed in an advisory, adaptive way, allowing for logical deletion and addition of tasks at runtime and override of plan parameters such as task execution time or preconditions [64]. Table 1 shows the list of the identified key features and their coverage in extended BPMN and TP.

**Table 1:** Coverage of key features in the extended business process model and notation (BPMN) and Task Planning (TP).

Feature	Extended BPMN	TP
Structured workflow definition	SESE^a^ restrictions	Built-in
Process modularity	Call activity	Dispatchable tasks or task hierarchy or subplans
Events	Timer or signaling events	Specialized task transition and state trigger events, among others
Parallel execution	Gateways	Execution type and concurrency mode
Task duration	BPMN extension	N/A^b^
Relative time constraints between tasks	BPMN extension	Built-in task-waits
Use of resources	Minimally defined	Minimally defined
Multiple tasks	Multiplicity marker	Repeatable tasks
Delays between task iterations	N/A	Repeat attribute “period”
Data integration	N/A, uses UML^c^	Capture data sets or subject’s proxy services
Overrides at the time of execution	Only add activities and events	Built-in (remove or add tasks, plan parameters, or subject preconditions)

^a^SESE: single entry, single exit.

^b^N/A: not applicable.

^c^UML: Unified Modeling Language.

### Step C: CR-BSI Case Study

#### Typical Process Patterns in Infection Treatment

The treatment of infections shares some common distinctive patterns that are typical of diseases caused by bacterial microorganisms. First, all types of infections require laboratory tests to determine the pathogen causing the infection in the first place to decide on the most effective antibiotic treatment. In addition, infections must be treated readily to avoid complications that could become life-threatening, following empirical evidence laid out in CGs even though laboratory results are not yet known. Furthermore, antibiotic treatment must follow strict administration rules to be effective, such as dosage, frequency, and duration. Finally, patients must be monitored throughout the entire process to quickly adjust the treatment in case problems arise. We represented some of these distinctive patterns of CR-BSI in TP to empirically check the suitability of TP for modeling infection CPs. The CR-BSI process patterns were originally elicited from the *JHH Antibiotic Guidelines* [15]. The *BPMN-based* CR-BSI process models [43,44] contain both standard BPMN flow objects and extended elements such as *task* and *edge duration*. For modeling the CR-BSI TP flows, we used the open-source draw.io tool, importing the TP-VML libraries [65]. Table 2 shows the correspondence of the extended BPMN process models from the studies by Zerbato [43] and Zerbato et al [44] with the TP process models depicted in this study.

**Table 2:** Correspondence between extended business process model and notation (BPMN) and Task Planning process models of catheter-related bloodstream infection.

Pattern	BPMN figure number	Description
1	5 [44]	ET^a^ (generic)
2	None	Determination of ET
3	3 [44]	*Staphylococcus aureus* treatment
4	4.1 [43]	CLT^b^ in coagulase-negative *Staphylococcus*

^a^ET: empiric treatment.

^b^CLT: catheter lock therapy.

#### Process Patterns in CR-BSI Treatment

We modeled the process patterns of CR-BSI (Textbox 1) using TP, maintaining the same logic and assumptions made in the study by Zerbato et al [44] when interpreting the JHH CGs.

**Process patterns of catheter-related bloodstream infection (CR-BSI).**

**Process patterns**

- *Empiric treatment* (ET): when suspicion of an infection exists, the clinical approach is to immediately start treating the patient with a wide-spectrum antibiotic following the empirically gained knowledge laid out in clinical guidelines (CGs) while laboratory tests are ordered in parallel to identify the causing microorganism. As soon as laboratory tests are available, the ET is revised and adjusted if required, either by changing its dosage or duration or replacing it with an organism-specific antibiotic for the concrete pathogen causing the infection.
- *Determination of ET*: this is typically a cognitive decision task that allows clinicians to select the best course of treatment based on CGs or clinician knowledge, clinical facts, and available culture results, if any.
- *Staphylococcus aureus treatment adjustment*: infection treatment usually involves strict management of temporal constraints as antibiotics need a specific administration pace and duration to be effective. During infection treatment, patient evolution and vital signs are continuously monitored to quickly adjust the originally prescribed therapy (eg, its dosage or duration).
- *Catheter lock therapy* (CLT): many infections are caused by the use of catheter implants in hospitalized patients, of which CR-BSI or *urinary tract infections* are typical examples. Intravascular catheters routinely develop microbial communities (biofilms) upon contact with environmental or skin pathogens. When such infections occur and the catheter cannot be removed as it can be counterproductive, antibiotic locks are recommended by CGs as complementary therapy so that both the systemic infection caused by the catheter and the area around the catheter are treated. This means that the *systemic therapy* must be repeatedly but not simultaneously applied with the CLT during a certain period. Both therapies use the same catheter as the vehicle for treatment, so the catheter becomes a shared resource that must be locked exclusively by each therapy task to prevent simultaneous use.

#### Pattern 1: Empiric Treatment

**Figure 2:**
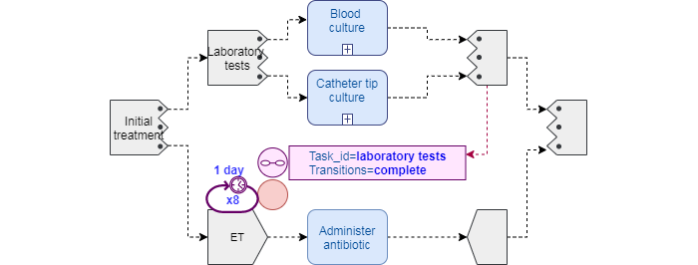
Catheter-related bloodstream infection empiric treatment (ET) pattern 1 using Task Planning Visual Modeling Language.

The empiric treatment pattern (Figure 2) illustrates the relevance of repeat loops and nondeterministic events in infection CPs. We represented a TP parallel *AND-all-paths* task group with 2 distinct branches: branch 1 represents the ordered set of *laboratory tests*, whereas branch 2 represents the *ET* administration. As more than one laboratory test is ordered, a second inner *AND-all-paths* task group is modeled to indicate that all cultures need to be completed to be able to interpret the results. Once they become available, a nondeterministic *task transition event* is fired, signaling the *ET* repeatable task, which has the *laboratory tests* transition event to the *complete* state as *repeat terminate condition*, ultimately interrupting the *ET* task.

This pattern was represented in extended BPMN [44] using deterministic events instead.

#### Pattern 2: Determination of Empiric Treatment

**Figure 3:**
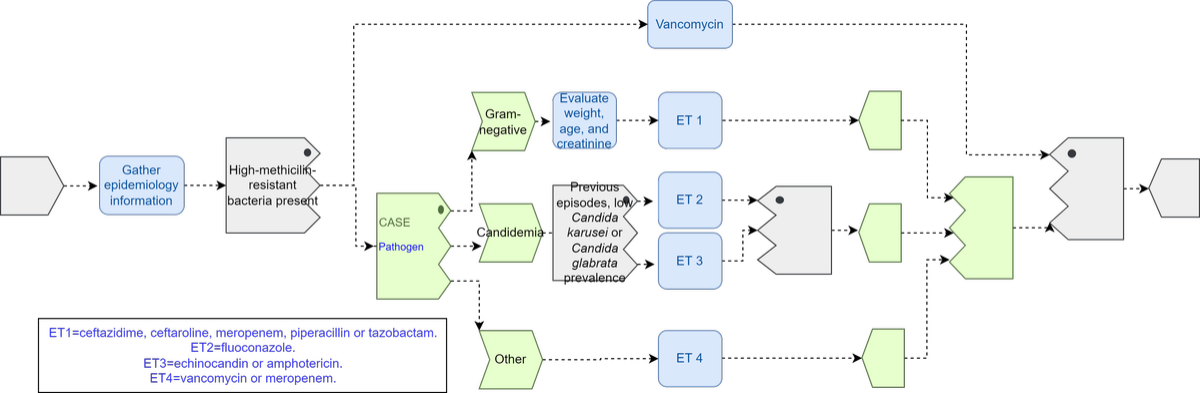
Catheter-related bloodstream infection determination of empiric treatment (ET) pattern 2 using Task Planning Visual Modeling Language.

This pattern shows the relevance of encapsulating decisions in rules, which was not possible in BPMN. In case of CR-BSI being suspected, the determination of the empiric treatment requires gathering epidemiology information from the hospital. If *methicillin-resistant* bacteria have high prevalence, a wide-spectrum antibiotic (*Vancomycin*) is usually recommended. Otherwise, a decision between different antibiotic treatments is taken (empiric treatment 1-4 for simplicity) based on the suspicion of a pathogen and on patient conditions. Figure 3 shows a representation using a *gate* task group for the binary decision *high–methicillin-resistant bacteria present*, which, if positive, includes a nested *case* task group with multiple exclusive treatment choices and an inner second binary *gate* to decide between *empiric treatment 2* and *empiric treatment 3*.

**Figure 4:**
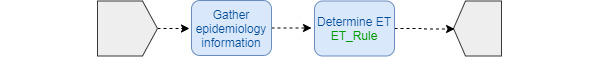
Catheter-related bloodstream infection determination of empiric treatment (ET) pattern 2 using Task Planning Visual Modeling Language with a Decision Language rule.

However, this pattern can be easily representable via a decision table, so we would rather model it in TP using a *DL* rule evoked from a *determine ET* task, which would encapsulate the knowledge associated with this decision in a single rule to be further maintained and evolved by knowledge experts, allowing for the separation of concerns (Figure 4).

#### Pattern 3: Staphylococcus aureus Treatment Adjustment

This pattern showcases the importance of overrides in dealing with uncertainty during the course of an infection treatment. The JHH CGs state the following: “Criteria for a 14-day course of therapy: patient is clinically stable; follow-up blood cultures drawn 2-4 days after the initial cultures are negative for *S. aureus*; the patient defervesces with 72 hours of initiation of effective antistaphylococcal therapy. All other patients should receive 4-6 weeks of therapy based on extent of infection” [15].

**Figure 5:**
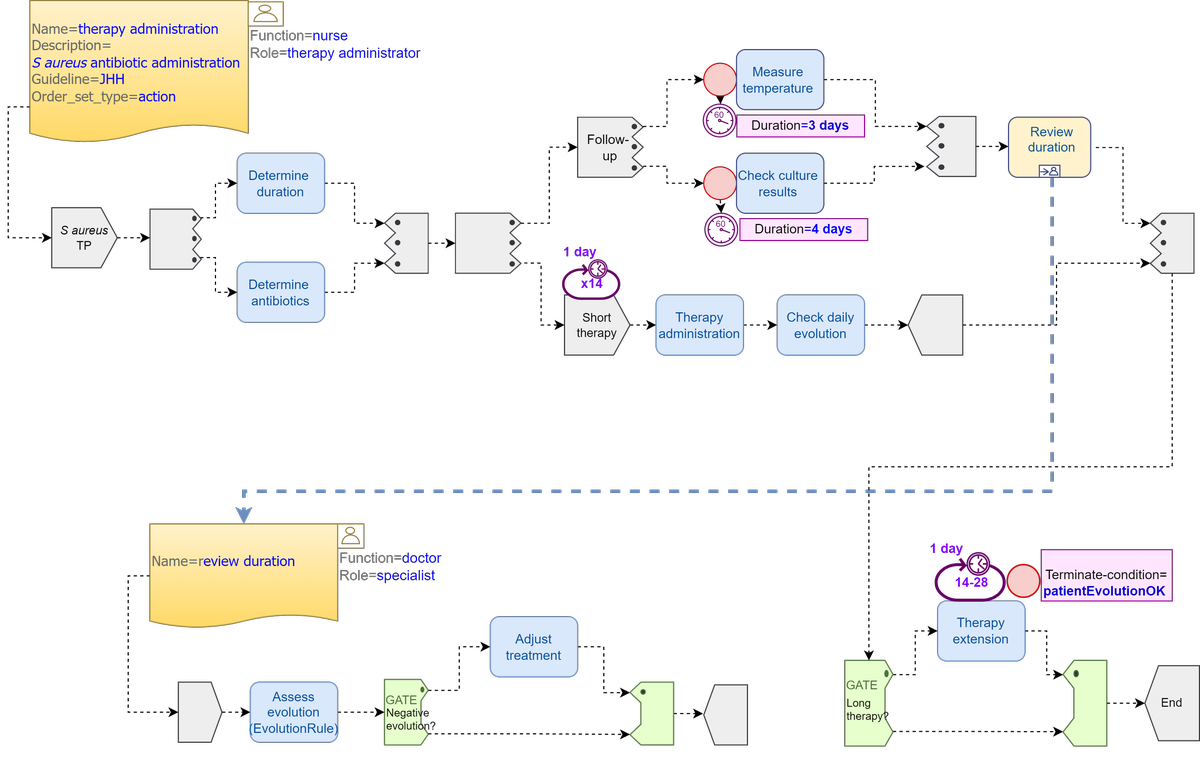
Catheter-related bloodstream infection *Staphylococcus aureus* (*S. aureus*) treatment adjustment pattern 3 (P3) using Task Planning (TP) Visual Modeling Language. JHH: Johns Hopkins Hospital.

Figure 5 shows a possible TP model using deterministic events, as in the study by Zerbato et al [44]. The decision *review duration* is a subplan that decides whether therapy duration should continue as planned or be prolonged to a minimum of 28 days and a maximum of 42 days. We used a parallel *and-all-paths* task group with 2 branches: the first branch is a parallel task group *follow-up* representing the *measure temperature* and *check culture results* timeline-driven tasks executed at day 3 and 4, respectively, after treatment start and the second branch is a repeatable task group that executes the initial 14-day *short therapy.* Once the *follow-up* branch ends, the *review duration* subplan is launched asynchronously to determine if the *short therapy* should be prolonged. When the *short therapy* ends, and only if a longer therapy was decided, a *therapy extension* repeatable task is launched until the minimum number of iterations (14) is reached. From that moment on, therapy can be interrupted through a *repeat-spec terminate condition* if a *state trigger* event signals positive patient evolution. The *therapy extension* cycle will end either when patient evolution is positive or, in the most extreme scenario, when the *repeat.upper* limit (28 in our case) is reached. Although this is a good example of how repeatable tasks are of great value for the representation of the course of treatment of infections, we had to perform some calculations with the days to faithfully represent the CPs. For simplicity, we assumed that the treatment was applied once daily but, in reality, antibiotics must be taken in strict time intervals expressed in hours rather than days. Alternatively, a DL rule can be introduced to assess patient evolution as part of the decision logic that determines either a short or a long therapy.

**Figure 6:**
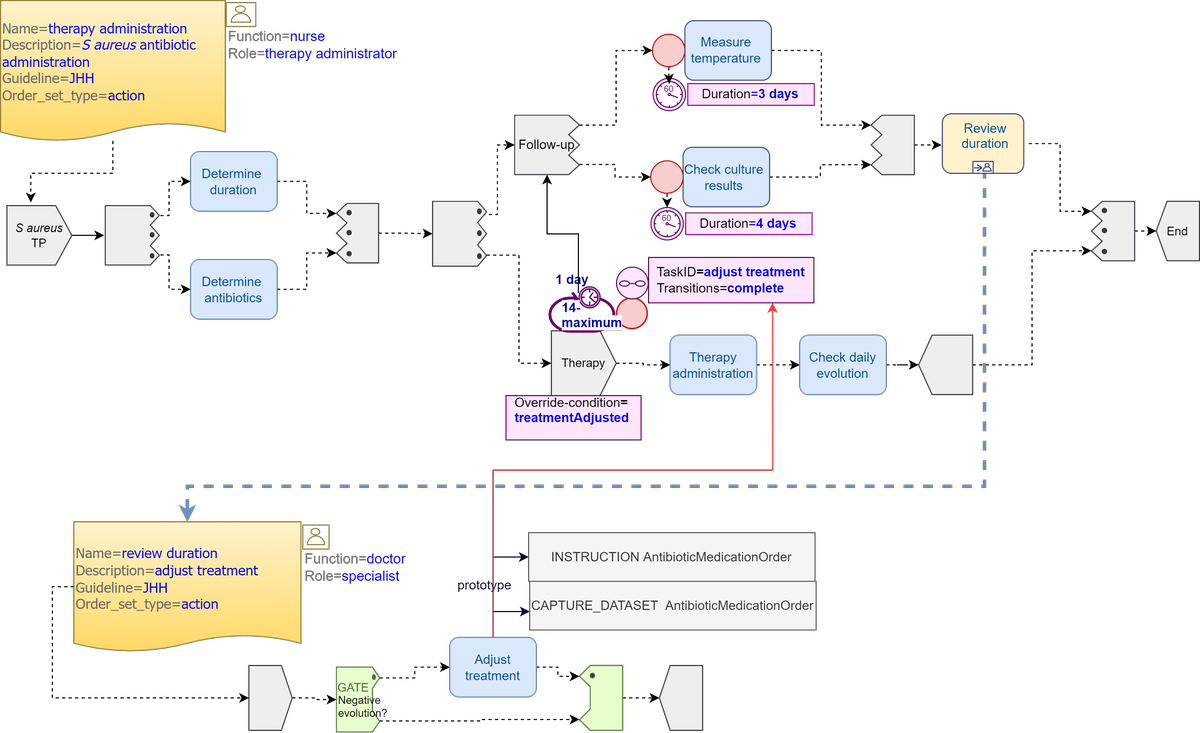
Catheter-related bloodstream infection *Staphylococcus aureus* (*S. aureus*) treatment adjustment pattern 3* using extended Task Planning (TP) Visual Modeling Language. JHH: Johns Hopkins Hospital.

The previous deterministic scenario could be improved, as shown in Figure 6. The main difference is that, in case treatment duration is prolonged by the *adjust treatment* task, a *capture data set* (form) would collect the new treatment information, update the EHR *subject’s proxy* relevant variables accordingly, and trigger a new repeat *override-condition* that would reset the repeat attributes to *repeat.lower=28* (instead of 14) and *repeat.upper=42*. However, this behavior has several implications that will be discussed in the following section.

#### Pattern 4: CLT

**Figure 7:**
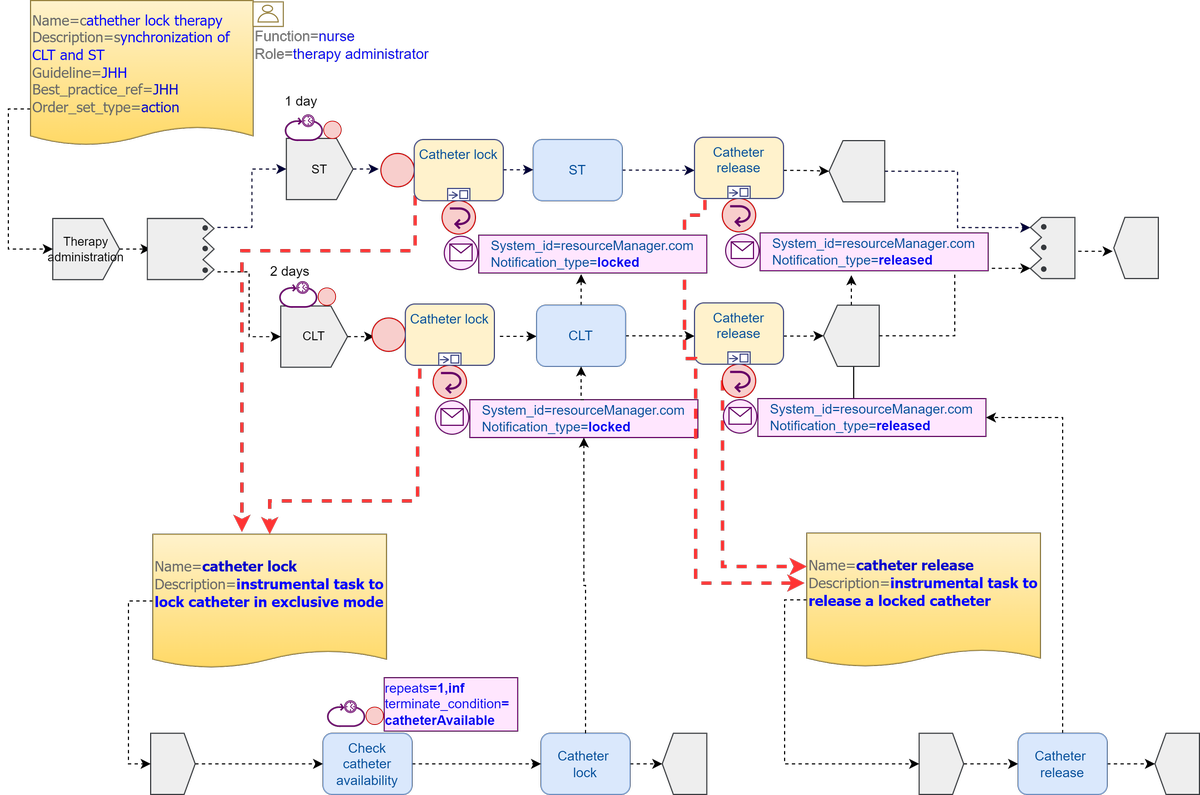
Catheter-related bloodstream infection catheter lock therapy (CLT) pattern 4 using Task Planning Visual Modeling Language. CG: clinical guideline; JHH: Johns Hopkins Hospital; ST: systemic therapy.

This pattern illustrates task synchronization when sharing a common resource in exclusive mode. CLT is a technique meant to reduce treatment failure that fills up a catheter with an antimicrobial agent and lets it dwell for a long-enough period. CLT must be administered through the same catheter used for ST, so task synchronization is essential to guarantee catheter availability. Owing to the lack of details in the JHH CGs regarding the alternation and frequency of CLT, we made a few assumptions: (1) both therapies are repeated within fixed but possibly different periods, (2) their timing is independent from each other, and (3) they take place at random but predictable times. With these premises, we modeled 2 repeatable parallel task groups: one including the *CLT* task and the other including the *ST* task. Both task groups first execute an instrumental *“catheter lock”* plan to issue an exclusive hold on the catheter as soon as it becomes available and end with a *catheter release*. The *catheter lock* includes a *repeatable* task group that checks catheter availability and ends as soon as the catheter becomes available, moment at which the catheter is locked, and a *callback notification* is sent to the calling task (either *ST* or *CLT*). Once the corresponding therapy task is executed, the trailing *catheter release* task is called to release the catheter (Figure 7).

**Figure 8:**
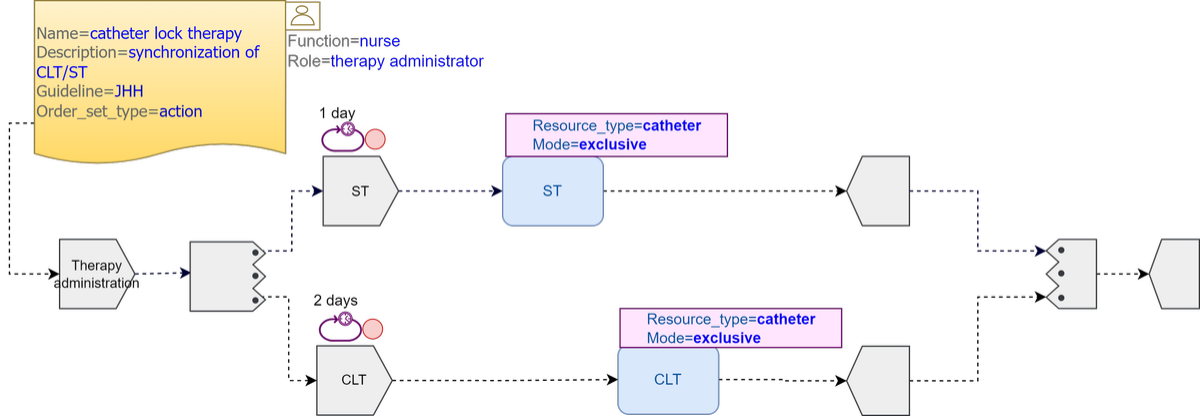
Catheter-related bloodstream infection catheter lock therapy (CLT) pattern 4* using extended Task Planning Visual Modeling Language. JHH: Johns Hopkins Hospital; ST: systemic therapy.

Ideally, this lock mechanism should be a *built-in* behavior in TP when a resource is defined as *exclusive*, greatly simplifying the process model as both the instrumental *catheter lock* and the *catheter release* task plans would not be necessary (Figure 8).

### Step D: Analysis and Possible Extensions

#### Overview

We were able to model typical process patterns of CR-BSI in TP (Figures 2-8) keeping the logic applied to the interpretation of the JHH CGs in the study by Zerbato et al [44] with no amendments or extensions to the current version of the TP standard. According to the 4 requirements for CP languages [58], the TP specification fulfills *requirement 1* with the openEHR architecture and foundation classes as well as many of the requirements *2, 3,* and *4* by means of parallel concurrency modes extensible with specific rules, *dispatchable* and *performable tasks* associated with *defined actions*, or temporal constraints between tasks using advanced events. In this study, we in fact analyzed the suitability of domain-specific features of *requirements 2 to 4* for infection CPs. However, in some cases, we depicted alternative process models using a different approach (eg, pattern 3* and pattern 4*) by proposing extensions that enhance the TP standard capabilities for modeling infection CPs, such as new annotation capabilities of tasks, enhanced cyclic constructs, new override behavior of the TP engine, new events, and enhanced resource management. To determine these potential TP extensions, we followed the methodology proposed in the study by Braun and Schlieter [58] for BPMN extensions, which we consider applicable for TP, consisting of 6 steps that extend the approach of Stroppi et al [66], resulting in an *equivalence* or *no equivalence* conclusion. A *no equivalence* conclusion can have 3 reasons: (1) the entire concept is missing (*extension concept*), (2) a relationship between 2 concepts is missing (*association between concepts*), or (3) attributes owned by a concept are missing (*property of a concept*).

Textbox 2 shows a summary of the main TP constructs used in the CR-BSI models. Models where an extended approach was proposed are denoted by a trailing *.

In the following sections, we analyze the most frequently used TP constructs and their possible improvements, which could lead to potential extensions of the standard.

**Main Task Planning (TP) constructs used in each use case.**

**TP construct and patterns in which it was used**

- And-all-paths parallel task group (TG): pattern 1, pattern 3, pattern 3*, pattern 4, and pattern 4*
- Repeat task or TG: pattern 1, pattern 3, pattern 3*, pattern 4, and pattern 4*
- Gate binary decision group: pattern 2, pattern 3, and pattern 3*
- Timeline event: pattern 3 and pattern 3*
- Asynchronous dispatchable task: pattern 3 and pattern 3*
- Task transition event: pattern 1 and pattern 3*
- Synchronous dispatchable task: pattern 4
- State trigger event: pattern 3
- System notification event: pattern 4
- Case decision group: pattern 2
- Decision Language rule: pattern 2*

#### Parallel Execution and Decision-making Constructs

The 4 TP *concurrency modes* associated with the parallel *execution type* cover a wide range of situations that might arise in actual clinical scenarios and were expressive enough for the representation of CR-BSI patterns. The *and-all-paths concurrency mode* was most frequently used, followed closely by the *gate decision group* construct*,* for single binary decisions. Occasionally, we used a *case decision group* in pattern 2, a cognitive diagnosis decision-making task that can be better represented as a single DL rule. Throughout the modeling process, we did not detect any need for improvements in the existing TP decision constructs or in parallel execution. However, the standard allows for the definition of rules if more sophisticated parallel behavior is required in complex scenarios.

#### Repeatable Task Constructs

**Figure 9:**
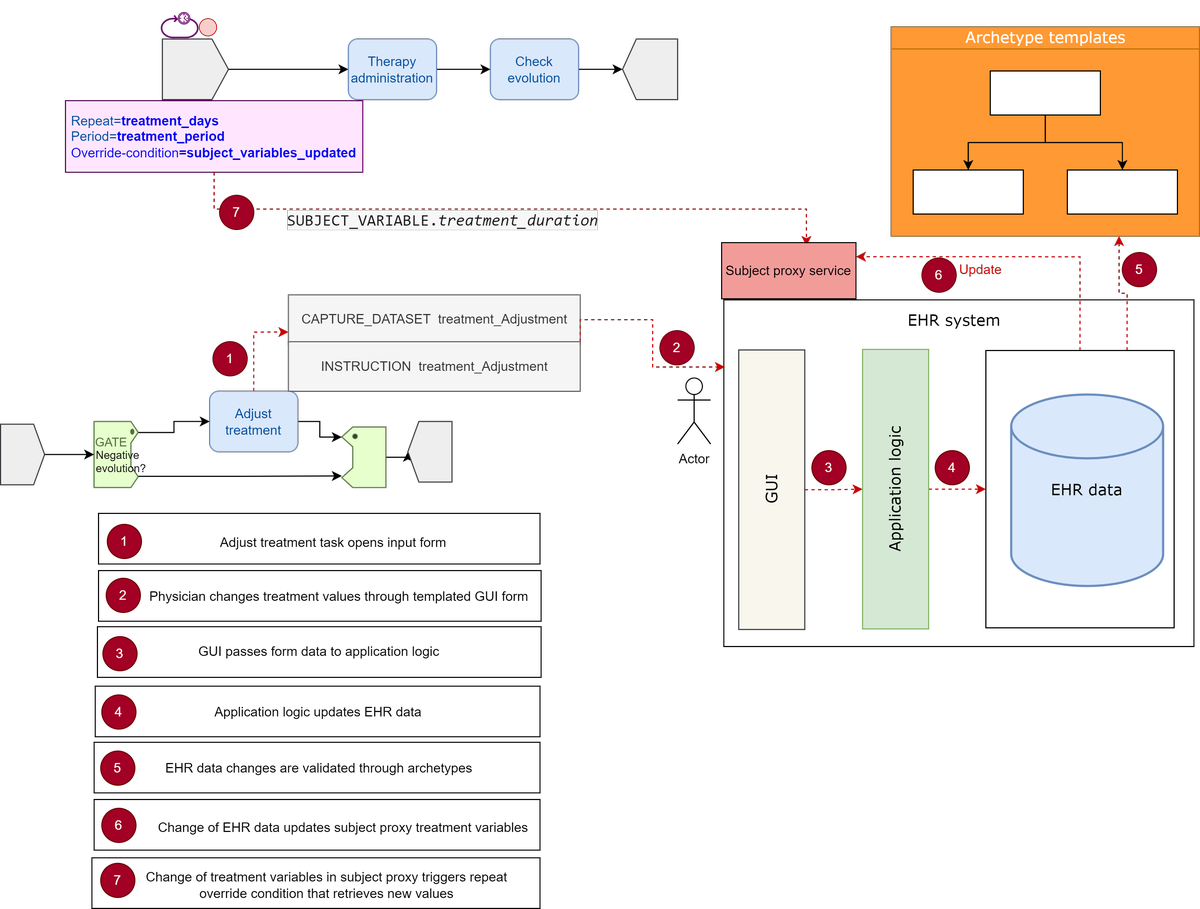
Possible mechanism of *Staphylococcus aureus* treatment adjustment pattern 3* with repeat override condition. EHR: electronic health record; GUI: graphical user interface.

The second most used TP construct was the task or task group *repeat*, widely used for medication plans, therapy administration, or monitoring of patients’ vital signs. Antibiotic treatments usually have the form *Ciprofloxacin 400 mg IV Q8H,* which can be represented in TP using the *repeat* attribute of a *plan item,* which unfolds copies of the successive antibiotic intakes in the TP execution engine. This attribute specifies a minimum and, optionally, a maximum number of iterations, a time interval or *period* between iterations, and a *terminate condition* evaluated as soon as the minimum number of iterations is reached before the execution of new iterations. The *terminate condition* can be any *plan definition* event exiting the *repeat* loop whenever event conditions are met. The TP *repeat* construct is a hybrid loop as it mimics both a *for* programming loop until the *repeat.lower* limit is reached and, after that, a *while* programming loop. This construct has proven to be essential for modeling CR-BSI CPs, which is why it could be further enhanced to achieve its full potential. For example, in pattern 3, treatment duration is adjusted *on the fly* when the patient does not evolve as expected—the treatment starts as a 14-day–long repeatable *short therapy* task and transitions smoothly to a 28- to 42-day *therapy extension*. The model is improved in the alternative pattern 3* to avoid the need to represent 2 distinct therapy tasks by proposing a mechanism to signal a repeatable task not to terminate but to modify its attributes while still running; that is, an override of the TP metadata model triggered by the process intrinsic logic. The main advantage of pattern 3* is a much simpler visual representation and a more powerful *repeat* construct with adaptive capabilities to respond to events not known at the time of process design. However, this behavior assumes a few things: first, the patient’s medication record is stored in the EHR and available to the TP engine; second, whenever treatment is adjusted, it first updates the EHR through a *capture data set*, generating a new medication instruction; third, there is some predefined mapping mechanism between the medication instruction and the *repeat* metadata, used by the TP engine upon the execution of the *adjust treatment* task (eg, taking the information in the *ORDER REF* class, which *“*represents a logical tracking reference to one ‘order’ in the real world*”* [14]); finally, the pattern 3* model would also require modifications in the TP abstract syntax, a new repeat *override condition* attribute in the *Task_Repeat* class to trigger the reset of the repeat attributes using the new treatment information in the *subject’s proxy* (Figure 9), and changes in the TP engine to keep internal state records of repeat iterations and dynamically update the materialized literal copies, unfolding pending iterations and considering the ones already consumed.

The *Task_Repeat* attributes would be automatically bound to the patient’s EHR medication record through a *defined action* associated with the task and its corresponding *prototype* based on its turn on a *medication archetype* containing a structured description of the therapy administration and time. When the *adjust treatment* task is performed, an *action* instance would be created from the *prototype* reflecting any divergences from the planned form of the task. To support this scenario, we suggested the extension of the *Task_Repeat* class, as outlined in Table 3.

The execution logic of our extended *Task_Repeat* class would be as follows: if *start-condition* is true and *repeat.lower* is >0, a loop is started of *1* to *repeat.lower* iterations (the *for* part of the *repeat* loop). This minimum number of iterations is executed unconditionally unless a *skip-condition* is met in any of the iterations. Only when iteration number *repeat.lower+1* is reached the rest of the conditions could apply; that is, an *override* or *terminate condition*.

**Table 3:** Proposed new attributes of Task Planning classes.

Class and attribute		Explanation	Possible values
**Task_Repeat**			
	Terminate-condition	Add more elaborated expressions or rules, possibly using DL^a^	N/A^b^
	Start-condition	New condition, expressed in a similar manner as the terminate-condition, would allow the first iteration to occur only if both the minimum iteration is not 0 and this condition evaluates to true	N/A
	Skip-condition	New condition, expressed in a similar manner as the terminate-condition, would skip the currently executing iteration if it evaluates to true by eliminating the materialized literal copy both in the “for” and the “while” parts of the loop.	N/A
	Override-condition	New condition, expressed in a similar manner as the terminate-condition, would allow for the overriding of Task_Repeat metadata at the time of execution	N/A
**Resource_Participation**			
	Category	Categorize resources with clinical process relevance	External or implant Medical device
	Mode	Exclusive or shared mode	Exclusive or shared
	State	State of clinically relevant resource	Allocated or locked Deallocated or unlocked
**Plan_Item**			
	minDuration	Minimum duration of Plan_Item	N/A
	maxDuration	Maximum duration of Plan_Item	N/A
	timeUnits	Units of time to express duration values	N/A
	durationUse	Specifies if the Plan_Item duration is information or also constraint	Informative or prescriptive

^a^DL: Decision Language.

^b^N/A: not applicable.

#### Resource Constructs

In infections, catheters are used as a vehicle for the administration of different therapies sometimes incompatible with each other, as we have seen in pattern 4 with *CLT* and *ST,* where we had to define 2 instrumental *catheter lock* and *catheter release* tasks, unnecessarily increasing flow complexity. In the alternative pattern 4* model, we eliminated the need for them by extending the *resource participation* class with new attributes to represent clinically relevant resources, the state of which can change at the time of execution owing to unexpected clinical happenings. This would require a *resource state machine* in the TP *materialized* model, similar to the TP engine *task state machine*, that would help improve the *resource perspective* of the TP standard, similar to how it was done for BPMN [59]. Accordingly, the TP engine should adopt a new specific behavior for resource allocation or deallocation at the time of execution. In the pattern 4* model, the *catheter* resource could be tagged as of *exclusive mode* so that the TP engine can exclusively allocate the resource to the executing task, implementing a *built-in* exclusive lock and release strategy to avoid *deadlocks*. Table 3 shows the proposed new attributes of the *Resource_Participation * class.

The proposed extensions affect the abstract syntax; that is, the TP standard metadata model and the TP engine logic. In addition, the EHR could document resources used in the patients’ medical procedures, especially those implanted that can cause or aggravate an infectious disease. For example, in CR-BSI or urinary tract infection, it is relevant to know if the catheter has been removed, irrigated, replaced, or salvaged to determine treatment duration.

#### Events

The TP *task-waits* allow for the introduction of dynamically determined delays between tasks, adding fundamental modeling capabilities through the use of specialized, *nondeterministic* events: *task transition*, *state trigger*, *callback, manual*, and *system notification*. The *task transition event* makes use of the TP engine *state machine* representing all possible states and transitions between tasks, which is essential for dynamically detecting task completion in infection treatment processes. The *state trigger event* signals changes in the values of global monitored variables representing patients’ vital signs. Together with the set of TP *deterministic events*, they provide advanced modeling capabilities in TP, greatly optimizing workflow execution times. We made use of a *task transition event* in pattern 1 or a *state trigger event* in pattern 3, which is consistent with the uncertainty and time-driven nature of the course of infection treatments. To complement these *nondeterministic* capabilities, we suggest the addition of a new *resource state event* to improve the *resource perspective* of TP, signaling tasks when resource allocation, deallocation, or exclusive locks or releases occur through a new *resource state machine* in the TP engine.

#### Temporal Constraints Between Tasks

The treatment of infections requires a rather strict regime of temporal constraints to optimize its effectiveness and, at the same time, limit antibiotic intake to the strictly necessary levels to avoid the development of antibiotic resistance. The BPMN *duration* extension, widely used in the study by Zerbato et al [44], does not currently exist in TP, and it has been replaced in our models with dynamic *nondeterministic* events, as in pattern 1, where a *task transition event* triggers a *repeat terminate-condition* to interrupt the *empiric treatment* loop. A *nondeterministic* event-driven model can speed up a treatment process (eg, when laboratory tests are ready earlier than expected, the pathogen-specific treatment can be started). Despite not being strictly required in our TP models, there could be other thinkable scenarios in which task *duration* could be useful, either as a constraint owing to regulations or in high-precision treatments. Arguably, medication records with duration information are already documented in the EHR; thus, a task *duration* attribute in the TP meta model could be seen as redundant. However, we think that a workflow task should have an intrinsic duration inherent to any piece of work which can be relevant for prescriptive tasks and overrides at the time of execution (eg, in a treatment adjustment) to dynamically change the number of antibiotic intakes. Consequently, we suggest adding optional *duration* attributes to the TP *Plan_Item* parent class (Table 3). The new attributes should be able to be overridden on demand at the time of execution and leave the corresponding accounting records for both audit and process-mining purposes. In addition, the concrete syntax (ie, the TP-VML graphical representation) could be enhanced with new annotation capabilities.

Furthermore, TP uses different *waits* to hold task execution: *task-*, *timer-*, and *callback-waits*. A list of events to be *waited on* can be specified in the *events*-*list* attribute of the *task*-*wait* class, both deterministic and nondeterministic. The *task-wait* ceases when any of the events in the *events-list* becomes true and is currently evaluated as a logical OR (cursive). We propose to add an attribute *event-list-relation* in the *task*-*wait* class with possible values *OR* and *AND* so that a task can wait on the simultaneous occurrence of more than one event, a typical behavior of *complex event processing* systems. However, this requires other possibly deep-going adaptations of the TP logic to evaluate the occurrence of multiple events within a given time window, so a thorough cost-benefit analysis should be performed on this specific feature. Furthermore, TP has an optional *event-relation* attribute in the *Task-wait* class for increased time granularity, intended to *“*allow a task to be specified as commencing before, with or after the triggering event (such as a meal)*”* [14]. In the case of deterministic events, this attribute could be further refined with a new *offset* attribute expressed in positive or negative time units relative to the triggering event as a *time quantifier*. As a *task*-*wait* can wait for more than one event, we would interpret the newly quantified event-relation as *transition task* t1 *to* the available *state* x *hours* before|after *the* events-list *via which* t1 task-wait *is ceased* in *AND* or *OR* logic; that is, all triggered events (*AND*) or just 1 (*OR*). The effect of this is to add or subtract a concrete amount of time (the *offset x*) to the firing time of the triggering event or events associated with the *task-wait*, causing the *task* to either be delayed or advanced. In the case of *nondeterministic* events, the *event-relation* attribute cannot have quantifiers, at least for the *before* operand, as the time of occurrence of such events is unknown, so the task can only become available immediately *before* or *after* the set of triggering events.

We have more generally analyzed TP time dependencies between tasks from a pragmatic point of view, checking if they meet the 13 Allen time interval operands [67], as summarized in Table 4. We focused on *performable tasks* as *dispatchable tasks* have their own synchronization mechanism through specific waits and events. We found that three pairs of the 6 symmetric Allen interval operands on task execution are naturally implemented in the TP standard: (1) *precedes* or *preceded by* is built upon the default behavior of the TP execution type *sequential*, which allows a task *t2* to become available when the predecessor task *t1* is completed plus an additional *task-wait* in the successor task *t2*; (2) *meets* or *met by* is a generalization of the previous case also built upon the execution type *sequential*, which allows a task *t2* to become immediately available for execution when the predecessor task *t1* is completed provided that no *task-waits* are included (this is the default behavior of *sequential* tasks); and (3) *starts* or *started by* is a temporal behavior achieved by modeling a parallel task group with tasks *t1* and *t2* and no *task-waits* included, so tasks *t1* and *t2* start at the same time.

The other 3 pairs of symmetric Allen interval operands plus the *equals* operand impose restrictions upon the tasks’ end or start times, always in parallel execution settings, and need more fine-grained time expressions, which is not always easy to achieve in the current version of the TP standard (Textbox 3).

Table 5 shows a list of the proposed TP extensions for modeling infection CPs, which we believe could also be of interest in other complex clinical settings. Most of the suggested extensions are labeled as either *“*property of a concept*,”* enriching already existing concepts, or as *“*extension concept*”* [58], mostly referring to a new behavior of the TP engine or materialized classes model.

Finally, Table 6 shows a comparison of the initially identified *key features* in extended BPMN, standard TP, and our proposed *extended TP* (represented in the TP* column) providing an overview of the possible areas of enhancement.

**Table 4:** Possible Task Planning (TP) representation of 13 Allen time relation operands.

Temporal relation	Graphic	Implementation in TP
Precedes (p) and preceded by (P)	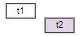	Sequential execution with “task-wait” or “period” in repeatable tasks
Meets (m) and met by (M)	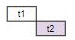	Sequential execution with no “task-wait” or “period” in repeatable tasks
Overlaps (o) and overlapped by (O)	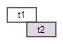	“Plan-time-origin” extension with new attribute “plan item”
Finishes (f) and finished by (F)	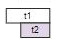	“Plan-item” class with new attribute “end time” or extended “task-wait to complete”
Contains (D) and during (d)	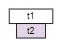	“Task-wait to complete” with triggering event “t2 completed”, plus time quantifier
Starts (s) and started by (S)	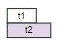	Parallel “task group” with no “task-wait” or with the same “task-wait”
Equals (e)	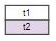	Parallel “and-all-paths” “task-group” with no “task-wait” and same task duration

**The remaining 3 pairs of symmetric Allen interval operands plus the equals operand.**

**The remaining operands**

- *Overlaps or overlapped by*: this relation can be modeled using a *parallel task* group (TG) and inserting *task-waits* either on the overlapping or the overlapped task. If the overlapping part of both tasks needs to be specified more accurately, this pattern should either include global timeline-specified *task-waits* for each task relative to the work plan start time or, ideally, time-lined *task-waits* with an offset relative to the first starting task (t1 or t2). However, this last scenario would require an extension of the *Plan_time_origin* enumeration class with a new attribute *plan-item* to specify a list of plan items of the parallel TG to be used as a relative reference for the timeline offset.
- *Finished by* or *finishes*: in this temporal pattern, parallel tasks *t1* and *t2* need to end at the same time. Generally speaking, an *and-all-paths parallel* task group could be used as it ends only when all TG branches are finished. However, the time to completion cannot be enforced as it depends on the longest executing branch, and it does not allow for the limitation of this behavior to 2 branches, for example. Thus, for a compulsory end time for *t1* and *t2*, a deterministic *end time* restriction should be applied to each task of the parallel TG. We could not find a way to implement this in the current Task Planning (TP) version, so a possible solution could require an extension of the *plan item* class with a new attribute *end time*: *ISO 8601 duration* or, alternatively, *task-waits* should apply to transition a task not only to the *available* state but also to any state or, at the very least for this specific scenario, to the *completed* state.
- *Contains* or *during*: this temporal relation states that *t2* must be started after *t1* has started and must end before *t1* ends (or vice versa). The start of *t2* after *t1* can be implemented as in the *overlaps* or *overlapped* case. However, the end of *t2* before *t1* is not that obvious to implement as *task-waits* are not an option because both tasks are already being executed by then. In the current TP version, we could not find a reasonable way to express that t2 *must end* x *units of time before the end of* t1 as a relative time constraint. To make it feasible, *task-waits* should apply to transition a task not only to *available* state but also to any state or, at the very least, to the *completed* state, as in the *finished by* or *finishes* pattern. In that case, a combination of a *task-wait to complete* having as a triggering event the end of *t2* with a time quantifier would allow for a more fine-grained expression of this pattern.
- *Equals*: this temporal constraint states that tasks *t1* and *t2* must start and end at the exact same time. For example, this can be achieved with a parallel TG with no task-waits and a specific restriction on each task duration. Thus, a possible solution would require the *duration* extension proposed previously.

**Table 5:** Summary of proposed Task Planning (TP) extensions for infection clinical pathways.

Extension	Class	Reason	Origin	Affects	Type
New “duration” attribute	Plan_Item	Informational, support Allen “equals” pattern	Pattern 1	AS^a^	PoC^b^
New repeat condition attributes	Repeat-spec	Enrich “repeat” construct	Pattern 3*	AS	PoC
New repeat “terminate-condition” value	Repeat-spec	Allow DL^c^ rule	Pattern 3	AS	PoC
New repeat behavior	TP engine	Override “repeat” metadata at the time of execution	Pattern 3*	L^d^	EC^e^
New resource attributes	Resource-participation	Extend resource perspective	Pattern 4*	AS	PoC
New resource allocation	TP engine	Implement behavior for exclusive resources	Pattern 4*	AS	EC
New resource transition event	Events	Detect resource state transitions	Pattern 4*	AS and CS^f^	EC
New resource state machine	TP engine	Specify transition between possible states of resources	Pattern 4*	AS	EC
New “event-list-relation” attribute	Task-wait	Allow for the specification of the logical relation between multiple triggering events (OR and AND)	Literature review	AS	PoC
New “offset” attribute	Task-wait	Allow for the delay or advancement of a deterministic event	Literature review	AS	PoC
New “resume-type” value	Resume-action	Allow for a rule as “resume-type”	Literature review	AS	PoC
New “end-time” attribute	Plan_Item	Support Allen “finished by” or “finishes” and “contains” or “during”	Allen	AS	PoC
New “start-time” attribute	Plan_Item_Origin	Start time of a plan item as reference for timeline in “overlaps”	Allen	AS	PoC
New “task-wait” behavior	TP engine	Task-waits on task transition to completed (“finished by” or “finishes” and “contains” or “during”)	Allen	L	EC

^a^AS: abstract syntax.

^b^PoC: property of a concept.

^c^DL: Decision Language.

^d^L: logic.

^e^EC: extension concept.

^f^CS: concrete syntax.

**Table 6:** Comparison of key features of extended business process model and notation (BPMN), Task Planning (TP), and extended TP.

Feature	Extended BPMN	TP	TP*
Structured workflow definition	Extension SESE^a^	Built-in	—^b^
Process modularity	Call activity	Dispatchable tasks in synchronous or asynchronous mode or subplans	New “resume-type” in synchronous dispatch
Events	Timer and signaling events	Specialized task transition and state trigger events	New “resource transition event”
Parallel execution	Gateways	Concurrency mode	—
Task duration	BPMN extension	N/A^c^	New task duration attributes
Relative time constraints between tasks	BPMN extension	Built-in “task-waits”	New “event-list-relation,” offset, “Plan_Item,” “plan-item-origin” attributes, and “TP engine” behavior
Use of resources	Minimally defined	Minimally defined	New resource attributes and “TP engine” behavior
Multiple tasks	Multiplicity marker	Repeatable tasks	New repeat conditions
Delays between iterations of looping tasks	N/A	Repeat attribute “period”	—
Data integration	N/A, uses UML^d^	“Capture data sets” and “subject’s proxy” services	Mapping between EHRs^e^ and TP metadata
Overrides at the time of execution	Few exceptions (add activities or events)	By design to remove or add tasks, plan parameters, and subject preconditions	On-the-fly override of model metadata (repeat) information

^a^SESE: single entry, single exit.

^b^None.

^c^N/A: not applicable.

^d^UML: Unified Modeling Language.

^e^EHR: electronic health record.

## Discussion

### Principal Findings

The results of our experiments show, on the one hand, the native suitability of TP for infection management and possibly for other complex clinical scenarios, confirming our initial hypothesis, and, on the other hand, the potential of the TP standard to simplify complex CPs by extending some of its native capabilities following the methodology for BPMN extensions laid out in the study by Braun and Schlieter [58], which we consider applicable to TP. The proposed TP extensions are mainly focused on increased synchronization between tasks, supporting Allen temporal relations, new dynamic behavior of repeat constructs, and enhancements in the resource perspective to emphasize the relevance of medical-associated devices, such as implanted catheters, in the course of infectious diseases. When applicable, we also proposed the use of complementary specifications such as DL.

In pattern 1, we saw how the rich set of nondeterministic TP events is better suited to represent uncertainty about task duration than the deterministic events used in the corresponding BPMN model, allowing for a dynamic response to unknown clinical happenings and, as a result, faster process execution. Nevertheless, we proposed to enhance the TP abstract syntax with new task duration attributes to allow for fine-grained fixed temporal constraints that could be of use in specific scenarios.

In the case of pattern 2, we initially modeled it in the depicted pattern 2 model as a cognitive decision-making task expressed as a multiple decision tree type of task, as done in BPMN. However, we proposed an alternative pattern 2* TP model to encapsulate the complexity of the cognitive decision-making task in a single rule instead. To this end, the TP standard is complemented by the DL specification, allowing for the evocation of DL rules from within a task or task group, which is not possible using BPM alone, resulting in simpler workflows and improved maintenance and evolution of task-related knowledge.

Pattern 3 highlights the need for a more advanced adaptive behavior of the TP execution engine to address new facts or clinical happenings that cannot be foreseen in advance in static BPM models, allowing for dynamic changes in the process logic at the time of execution. The pattern 3 TP model mimics the corresponding BPMN model, with 2 distinct cyclic therapy tasks. However, in the alternative pattern 3* model, we proposed extending TP to enhance the override capabilities of the *repeat* loop, which is widely used in infection CPs to represent, for example, cyclic medication tasks. Dynamic overrides were not available in BPMN, resulting in an unnecessary increase in the workflow complexity.

Finally, pattern 4 shows the synchronization between 2 tasks that cannot be executed simultaneously through the same device; in our case, a catheter. This pattern could not be represented in BPMN owing to its complexity. We could represent it easily in the pattern 4 TP depicted model using *out-of-the-box* TP constructs by modeling 2 instrumental tasks for synchronization purposes complemented with the rich set of nondeterministic TP events. However, in the alternative pattern 4* model, we showed how enhancing the TP standard with a resource extension could greatly simplify the pattern 4 representation by adding new attributes to the *Resource_Participation* class and proposing both a new nondeterministic *resource_transition* event and a new *state machine* in the TP engine. The proposed TP extension could be generally used to represent medically relevant devices such as catheters, mechanical ventilators, or any other disease-associated devices, thus improving the TP native *resource perspective*.

Furthermore, we theoretically analyzed other possible extensions that could be of use in infection management and other clinical scenarios, such as adding *complex event processing* capabilities to the standard, an enhanced *task*-*wait* condition logical expression using the newly proposed *event-list-relation* and *offset* attributes, and a new *Resume_type* in a task synchronous dispatch. Finally, we examined the TP temporal restrictions between tasks from a theoretical point of view, analyzing how the TP standard addresses the 13 Allen temporal relations and proposing extensions to support 3 of the 6 symmetric Allen relations plus the *equals* temporal operator. However, the proposed temporal extensions must be weighed against their implementation costs as they require an enhanced *task*-*wait* logic for a task to transition not only to the *available* state but also to the *completed* state, as well as new *time reference* attributes to be able to express more sophisticated time constraints between tasks.

### Strengths and Limitations

The proposed extensions provide a more consistent approach to dealing with uncertainty, thereby improving overall workflow efficiency and duration by adding dynamic response capabilities during workflow execution. They also acknowledge the importance of clinically relevant resources in the course of infection treatment, improving their visibility and clinical role.

A possible limitation is that many of the proposed extensions require changes in the abstract syntax and the behavior of the TP engine; for example, in pattern 3* to dynamically monitor and reset the *repeat* loop count. These changes in the standard should be weighed against the expected benefits. In addition, the use of rules within CPs to represent knowledge-intensive tasks requires further definition of the DL specification, which is still under development. Furthermore, the use of resources in CPs should be synchronized with the resource information defined in the EHR through specific mechanisms. Finally, the overall suitability of the standard for other complex clinical domains should be further assessed in future studies addressing the specific domain constraints.

### Future Directions

Future work will focus on analyzing the DL specification [50] in relation to TP, a new *openEHR* formalism for evoking rules from TP expressed using the *openEHR Expression Language* [68] and based on the *openEHR Basic Meta Model* [51]. We will also analyze the new OMG *BPM+ Health* initiative aiming to promote the use of the 3 OMG standards—BPMN, Case Management Model and Notation, and Decision Model and Notation—in health care domains [69].

## Abbreviations

BPM: business process management
BPMN: business process model and notation
CEN: European Committee for Standardization
CG: clinical guideline
CLT: catheter lock therapy
CMMN: Case Management Model and Notation
CP: clinical pathway
CR-BSI: catheter-related bloodstream infection
DL: openEHR Decision Language
DMN: Decision Model and Notation
EHR: electronic health record
GLIDE: Graphic Language for Interactive Design
HL7: Health Level 7
HRID: human-readable identifier
ISO: International Organization for Standardization
JHH: Johns Hopkins Hospital
OMG: Object Management Group
SHACL: Shapes Constraint Language
SPIN: SPARQL Protocol and RDF Query Language Inferencing Notation
SPARQL: Simple Protocol and RDF Query Language
ST: systemic therapy
TP: openEHR Task Planning
TP-VML: Task Planning Visual Modeling Language
YAWL: Yet Another Workflow Language
